# Trends in Rural and Urban Cigarette Smoking Quit Ratios in the US From 2010 to 2020

**DOI:** 10.1001/jamanetworkopen.2022.25326

**Published:** 2022-08-03

**Authors:** Maria A. Parker, Andrea H. Weinberger, Emma M. Eggers, Erik S. Parker, Andrea C. Villanti

**Affiliations:** 1 Department of Epidemiology and Biostatistics, Indiana University School of Public Health, Bloomington, Indiana; 2 Ferkauf Graduate School of Psychology, Yeshiva University, Bronx, New York; 3 Department of Psychiatry and Behavioral Sciences, Albert Einstein College of Medicine, Bronx, New York; 4 Department of Epidemiology and Population Health, Albert Einstein College of Medicine, Bronx, New York; 5 Department of Health Behavior, Society and Policy, Rutgers School of Public Health, Piscataway, New Jersey; 6 Department of Health Behavior, Society and Policy, Rutgers Center for Tobacco Studies, New Brunswick, New Jersey

## Abstract

This cross-sectional study examines self-reported smoking patterns of adult participants in the US National Survey on Drug Use and Health (NSDUH).

## Introduction

Cigarette smoking prevalence is higher in rural than urban US communities. This disparity has increased over time.^1^ People in rural vs urban areas are more likely to die prematurely,^2^ which has been associated with reduced health care access and smoking cessation barriers.^3^ Lower smoking cessation rates^4^ could also be factors in increased morbidity and mortality burden in rural residents.^2^ Herein, we estimated trends in cigarette quit ratios for adults (≥18 years) in rural and urban areas from 2010 to 2020.

## Methods

Publicly available, deidentified data were obtained from 2010-2020 National Survey on Drug Use and Health (NSDUH). Adults who had smoked 100 or more cigarettes were included in the analyses. This cross-sectional study followed the STROBE reporting guideline and was considered to be non–human participant research by Indiana University Institutional Review Board.

Rurality was defined using US Office of Management and Budget Rural-Urban Continuum Codes. For 2010-2014 NSDUH, definitions were based on 2003 metropolitan or nonmetropolitan statistical area county-level groupings; for 2015-2020 NSDUH, updated 2013 groupings were used. Lifetime cigarette smoking was defined as having smoked at least 100 cigarettes in a lifetime, current smoking as 1 or more cigarettes in past month, and former smoking as no cigarettes in the past year. Overall and annual quit ratios were estimated as proportions of former smokers among lifetime smokers.

Current smoking and quit ratios were estimated by rural or urban residence annually. Weighted, stratified logistic regression models tested linear time trends in quit ratios by rural or urban residence using year as a continuous variable. Models were unadjusted and then adjusted for sex, race and ethnicity, educational level, and income. Adjusted models, including year by rurality and year by age, were estimated separately to explore differential rural or urban and age time trends in quit ratios using the R survey package. α = .05 indicated significance.

## Results

Of the 161 348 lifetime cigarette smokers analyzed, 54 080 (33.5%) were former smokers. Participants ranged in age (14.2% were 18-25 years; 15.9%, 26-34 years; 25.3%, 35-49 years; 44.6%, ≥50 years) and included 48.2% women and 51.8% men; 64.9% White individuals; and 35.8% individuals with more than $75 000 annual income.

**Figure 1.**
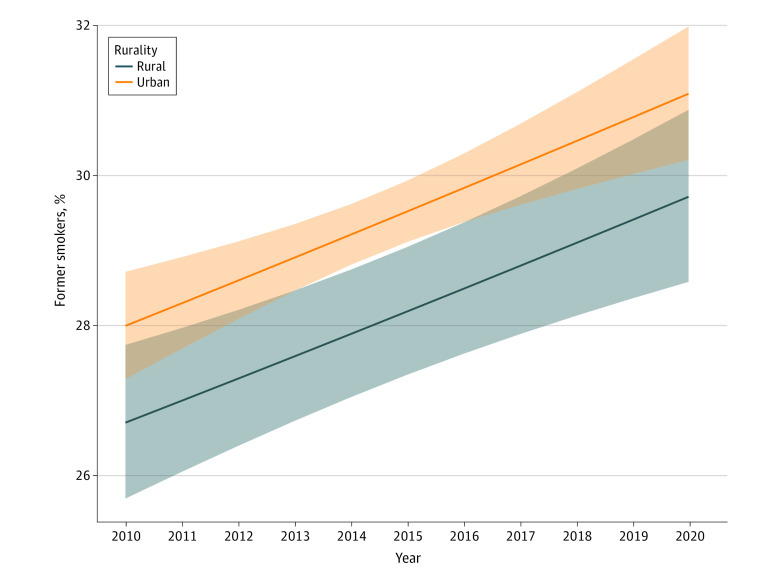
Smoking Quit Ratios for Individuals in Rural vs Urban Areas From 2010 to 2020

**Figure 2.**
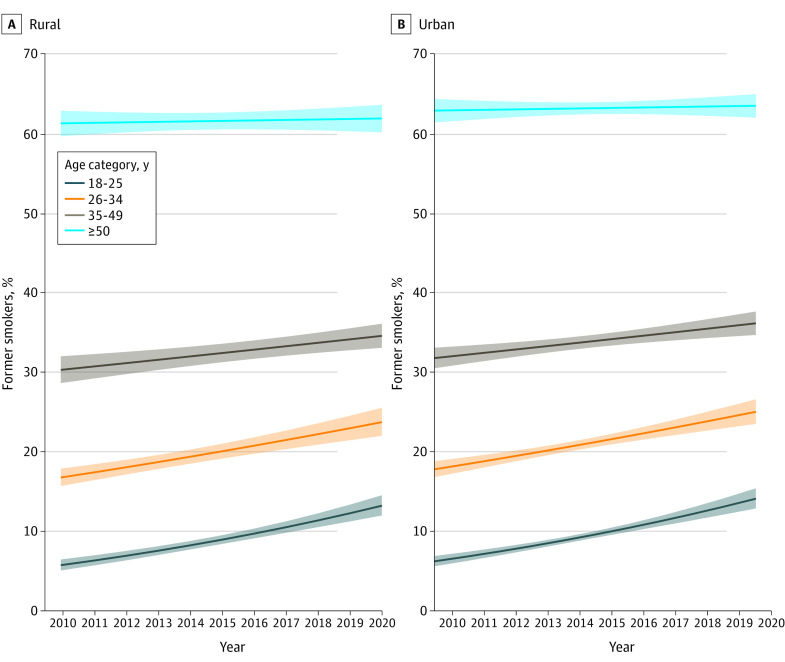
Smoking Quit Ratios by Age for Individuals in Rural vs Urban Areas From 2010 to 2020

In 2020, current smoking prevalence was higher in rural (19.2%; 95% CI, 16.9%-21.7%) than urban areas (14.4%; 95% CI, 13.3%-15.5%; *P* < .001), whereas quit ratios were similar in rural (52.9%; 95% CI, 48.3%-57.4%) and urban areas (53.9%; 95% CI, 51.4%-56.5%; *P* = .70). From 2010 to 2020, odds of quitting were lower in rural vs urban areas (odds ratio [OR], 0.85 [95% CI, 0.81-0.88; *P* < .001]; adjusted OR [AOR], 0.93 [95% CI, 0.89-0.98; *P* = .008]). Quit ratios increased over time (OR, 1.03 [95% CI, 1.03-1.04; *P* < .001]; AOR, 1.01 [95% CI, 1.01-1.02; *P* < .001]) (Figure 1), and no significant interaction was observed between rural or urban residence and time (χ^2^ = 0.027; *P* = .89). However, the interaction between age and time was significant (χ^2^ = 75.90; *P* < .001) (Figure 2); odds of quitting were still lower in rural vs urban areas (AOR, 0.93; *P* = .006).

## Discussion

Findings from 2010-2020 NSDUH data support a persistent rural-urban disparity. Higher smoking prevalence and lower quit ratios were observed in rural vs urban areas after adjusting for sociodemographic characteristics. These results may reflect an earlier stage of motivation to quit^5^ and reporting of higher nicotine dependence and heaviness of smoking among rural vs urban residents.^6^ Rural residents may also face structural barriers to cessation services, including lower rates of insurance coverage and fewer available health care practitioners,^3^ which warrant future research.

A study strength is its high external validity. Limitations include its cross-sectional design, although we used multiple years of data, and potential measurement error from self-reported data.

Intervention at the clinical setting, health system, or population level might improve reach and sustainability of cessation services for rural, especially older, residents. Leveraging existing quit lines and telehealth solutions may minimize barriers to accessing cessation services.^3^

## References

[1] Doogan NJ, Roberts ME, Wewers ME, . A growing geographic disparity: rural and urban cigarette smoking trends in the United States. Prev Med. 2017;104:79-85. doi:10.1016/j.ypmed.2017.03.011 28315761 PMC5600673

[2] Mansfield CJ, Wilson JL, Kobrinski EJ, Mitchell J. Premature mortality in the United States: the roles of geographic area, socioeconomic status, household type, and availability of medical care. Am J Public Health. 1999;89(6):893-898. doi:10.2105/AJPH.89.6.893 10358681 PMC1508666

[3] Talbot JA, Williamson ME, Pearson K, . Advancing Tobacco Prevention and Control in Rural America. National Network of Public Health Institute; 2019:75.

[4] American Lung Association. Rates by state. Accessed November 30, 2021. https://www.lung.org/research/trends-in-lung-disease/tobacco-trends-brief/rates-by-state

[5] Wewers ME, Stillman FA, Hartman AM, Shopland DR. Distribution of daily smokers by stage of change: current population survey results. Prev Med. 2003;36(6):710-720. doi:10.1016/S0091-7435(03)00044-6 12744915

[6] Baker TB, Piper ME, McCarthy DE, ; Transdisciplinary Tobacco Use Research Center (TTURC) Tobacco Dependence. Time to first cigarette in the morning as an index of ability to quit smoking: implications for nicotine dependence. Nicotine Tob Res. 2007;9(suppl 4):S555-S570. doi:10.1080/14622200701673480 18067032 PMC2933747

